# Use of Cross-Sectional Imaging Body Composition Assessment to Predict Pancreas Transplant Outcomes

**DOI:** 10.3389/ti.2025.15000

**Published:** 2025-10-01

**Authors:** Colin Snook, Tim Ziemlewicz, Glen Leverson, Sandesh Parajuli, Didier Mandelbrot, David P. Al-Adra, Dixon B. Kaufman, Jon S. Odorico, David D. Aufhauser

**Affiliations:** ^1^ School of Medicine and Public Health, University of Wisconsin, Madison, WI, United States; ^2^ Department of Radiology, University of Wisconsin, Madison, WI, United States; ^3^ Department of Surgery, University of Wisconsin, Madison, WI, United States; ^4^ Department of Internal Medicine, University of Wisconsin, Madison, WI, United States

**Keywords:** body mass index, hazard ratio, pancreas transplant alone, post-transplant diabetes mellitus, skeletal muscle index

Dear Editors,

Obesity has traditionally been a relative contraindication to pancreas transplantation due to concerns about the association between obesity and elevated peri-operative risk as well as development of post-transplant insulin resistance [1]. However, studies have shown equivalent outcomes between overweight and non-overweight simultaneous pancreas and kidney transplant (SPK) patients based on the low body mass index (BMI) cutoff of 28 [2, 3]. The impact of more pronounced obesity, and how that is classified, on pancreas transplant outcomes remains unknown. Although easy to calculate, BMI does not account for differences in fat distribution between ethnicities, genders, sex, age, and genetic backgrounds. Cross-sectional imaging allows more granular evaluation of a patient’s body composition, including direct measurement of visceral and subcutaneous adiposity and assessment of associated sarcopenia. Individual variation in adipose distribution may be particularly important to assessing risk in pancreas transplant recipients because of the prominence of visceral adiposity in the metabolic syndrome and the development of insulin resistance [4].

Studies incorporating CT-based metrics have associated visceral adiposity with poor outcomes following many types of surgery, including liver and kidney transplantation [5–7]. The only prior study assessing CT metrics of body composition in pancreas transplantation described a protective effect of adipose tissue on the risk of postoperative complications but was limited by a small sample size of both obese (n = 6) and overall (N = 40) patients [8]. Therefore, the impact of body composition on pancreas transplant outcomes remains unknown.

We performed a retrospective, single-center study analyzing the preoperative CT scans of adult, first-time pancreas transplant recipients between 2012–2020 to determine the relationship between visceral adiposity, sarcopenia, and post-transplant outcomes. Visceral adiposity was defined separately in men and women as the quartile of patients with the highest visceral adipose tissue-to-subcutanoues adipose tissue ratio (≥0.84 in men and ≥0.51 in women). Sarcopenia was defined similarly as the quartile of patients with the lowest SMI (<51.2 cm^2^/m^2^ in men and <43.1 cm^2^/m^2^ in women). Detailed Materials and Methods can be found in the Supplementary Data.

The study included 204 pancreas transplant recipients, 146 (71%) with type 1 diabetes mellitus (T1DM) and 58 (29%) with type 2 diabetes mellitus (T2DM). The mean follow-up was 4.9 ± 2.4 years. Patients with visceral adiposity were older (50.7 ± 10.0 vs. 46.6 ± 10.0, p = 0.01) and had a higher incidence of T2DM (20/52 vs. 38/152, p = 0.046). Fifteen patients (7%) met criteria for both visceral adiposity and sarcopenia. Patients with visceral adiposity received organs from younger donors (23.3 ± 11.9 vs. 27.6 ± 25.6, p = 0.02). Donor sex, age, donation after circulatory death status, pancreas donor risk index, cold ischemic time, hospital length of stay, readmission within 30 days, and incidence of delayed graft function were similar between the groups (Supplementary Table S1).

Visceral adiposity was associated with decreased patient survival post-transplant (p = 0.04, Figure 1A) but not decreased pancreatic graft survival (p = 0.25, Figure 1B). Sarcopenia did not impact patient (p = 0.24) or pancreatic graft survival (p = 0.49). Among SPK recipients, sarcopenia was associated with decreased kidney allograft survival (p = 0.03, Figure 1C). Post-transplant diabetes mellitus (PTDM) was not impacted by either exposure (p = 0.49 for visceral adiposity and p = 0.53 for sarcopenia).

**FIGURE 1:**
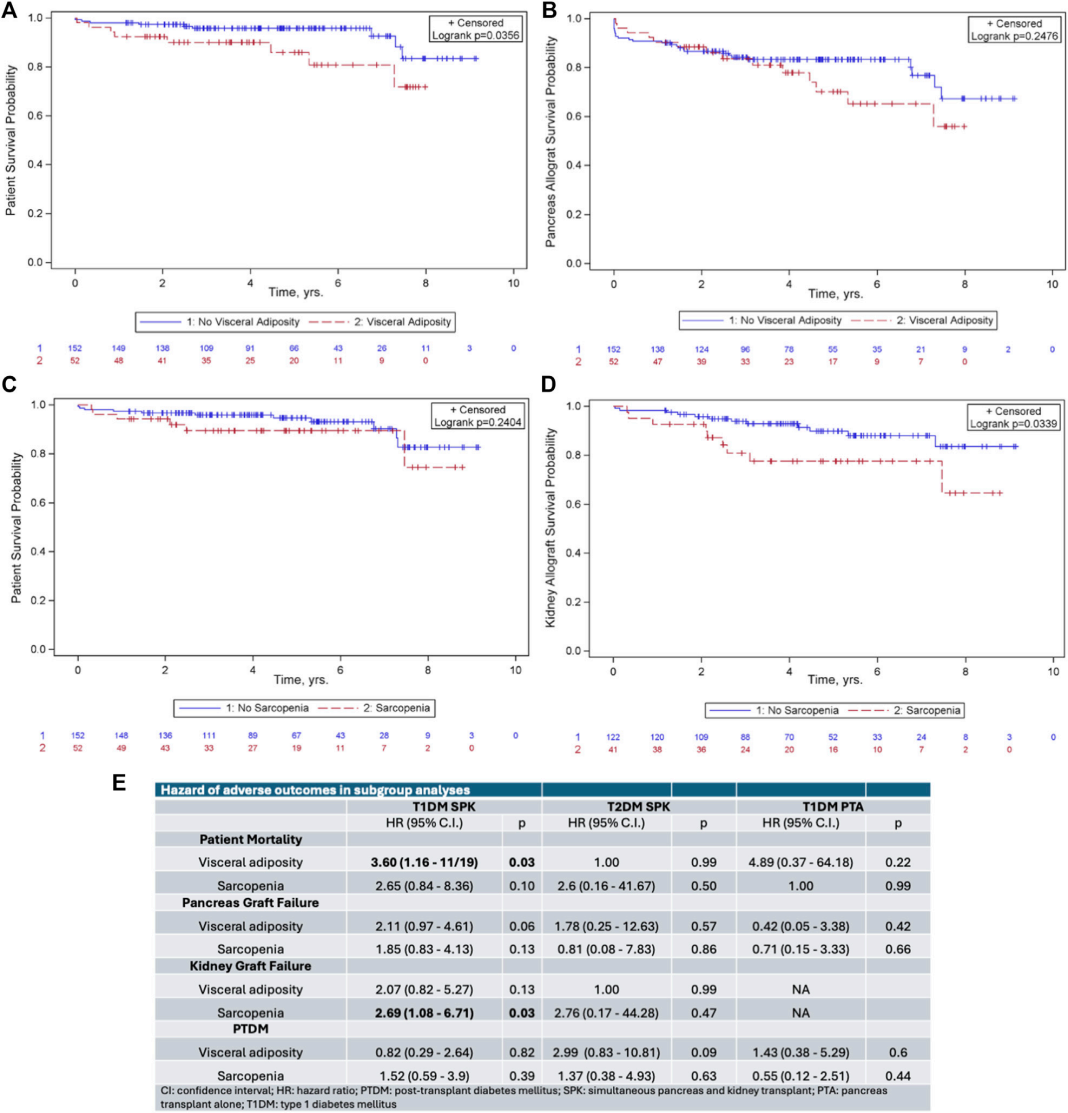
Kaplan-Meier survival curves demonstrating the impact of **(A)** visceral adiposity on patient survival, **(B)** visceral adiposity on pancreas allograft survival, **(C)** sarcopenia on patient survival, and **(D)** sarcopenia on kidney allograft survival. **(E)** Subgroup analysis of hazard ratios of adverse outcomes following pancreas transplantation.

Because the end-organ effects of diabetes are different in patients based on type of diabetes and in those receiving SPK versus pancreas transplant alone (PTA), we hypothesized that body composition may impact these patients differently. We therefore performed subgroup analysis based on type of diabetes and transplant type. In SPK recipients with T1DM, visceral adiposity remained associated with decreased patient survival (hazard ratio [HR] 3.60, p = 0.03, Figure 1E) and sarcopenia remained associated with decreased kidney allograft survival (HR 2.69, p = 0.03, Figure 1E). Neither visceral adiposity nor sarcopenia impacted outcomes in SPK recipients with T2DM or in PTA recipients with T1DM.

This eight-year experience represents the largest examination of the impact of body composition on pancreas transplant outcomes and has two principal findings. First, visceral adiposity is associated with decreased patient survival following pancreas transplant. Second, sarcopenia is associated with worse kidney allograft survival in SPK recipients. In subgroup analysis, these findings were restricted to SPKs recipients with T1DM.

The general association of visceral adiposity and sarcopenia with worse outcomes following pancreas transplant is consistent with results reported following other varieties of surgery. More surprising is the lack of impact of visceral adiposity on either pancreatic allograft survival or PTDM given the well-reported correlation between visceral adiposity, metabolic syndrome, and insulin resistance and the previously reported association of visceral adiposity and PTDM in kidney transplant recipients [9]. The finding that the adverse impacts of visceral adiposity and sarcopenia were confined to recipients with T1DM suggests that the impact of body composition may vary based on type of diabetes. This conclusion is consistent with recent work suggesting that genetic subtypes of adipose distribution have a differential impact on T2DM risk [10].

This analysis has several limitations. First, there are not consensus definitions of visceral adiposity and sarcopenia in this patient population. We attempted to mitigate this limitation by analyzing our data with different thresholds including median values of each sex and other published criteria and saw only minor differences. Second, the sample size is relatively small and may be underpowered for subtle differences. Finally, the study is retrospective and only captures results from patients who were robust enough to complete the pancreas transplant evaluation process. Patients with severe visceral adiposity and/or sarcopenia may have been excluded through other related criteria, including BMI cutoffs and frailty assessments.

This study underscores that visceral adiposity and sarcopenia adversely impact pancreas transplant outcomes. Evolving technology, including the use of artificial intelligence to rapidly and objectively calculate metrics of body composition, can facilitate assessment of these variables in pancreas transplant candidate evaluation and may help define more concrete thresholds. Moreover, these metrics can trigger effective steps to help patients with visceral adiposity or sarcopenia reduce their post-transplant risks through measures like anti-obesity medication and proactive physical rehabilitation. Shifting evaluation criteria toward assessment of body composition instead of BMI might allow more patients to qualify for pancreas transplantation while safeguarding excellent post-transplant results.

## Data Availability

The raw data supporting the conclusions of this article will be made available by the authors, without undue reservation.

## Abbreviations

BMI, body mass index; HR, hazard ratio; PTA, pancreas transplant alone; PTDM, post-transplant diabetes mellitus; SMI, skeletal muscle index; SPK, simultaneous pancreas and kidney transplant; T1DM, type 1 diabetes mellitus; T2DM, type 2 diabetes mellitus.
