# Supplementary material for: Use of Cross-Sectional Imaging Body Composition Assessment to Predict Pancreas Transplant Outcomes
Source: Transpl Int. 2025 Oct 1;38:15000. doi: 10.3389/ti.2025.15000 (PMC12521003; doi:10.3389/ti.2025.15000)
Supplement: Supplementary file 1 [file DataSheet1.docx]

**Supplemental Data**

**Materials and Methods**

*1. Data Source*

Data were retrospectively collected from all pancreas transplants performed at the University of Wisconsin between July 1, 2012, and June 30, 2020, with follow-up through March 30, 2022. Data were obtained from the Wisconsin Allograft Recipient Database. The end date was selected to ensure ≥1 year follow-up for all patients included in the study. Adult (≥18 years) first-time pancreas recipients were included in the analysis. Pediatric recipients (<18 years), recipients of prior transplants, and recipients without pre-operative CT scans within 24 months of the transplant were excluded. The study was approved by the Health Sciences Institutional Review Board at the University of Wisconsin (protocol #2014-1072). All research was conducted in accordance with both the Declarations of Helsinki and Istanbul.

*2. Immunosuppression*

Pancreas transplant recipients at our institution generally received antibody-based lymphocyte-depleting induction with either anti-thymocyte globulin or alemtuzumab at the discretion of the transplantation surgeon, with selected patients receiving non-depleting induction with basiliximab. Maintenance immunosuppression consisted of tacrolimus and mycophenolate mofetil. Steroid withdrawal or continuation was at the discretion of the operative surgeon. Tacrolimus trough goals were 8-10 ng/mL for the first 12 months.

*3. Analytic Approach*

Recipients were classified as having T1DM or T2DM as previously described (15). Pre-operative CT scans were analyzed by one author (CS) using 3D Slicer (16) and results validated by an attending radiologist (TZ). Adipose tissue was identified by using a voxel density threshold of -190 Hounsfield Units (HU) to -30 HU. Separate measurements were taken to assess Visceral Adipose Tissue (VAT), Subcutaneous Adipose Tissue (SAT), and Total Adipose Tissue (TAT) at the midlevel of the L4 vertebra by applying a user-drawn region of interest (ROI) around anatomical features specific to SAT, VAT, and TAT. SAT was defined as adipose tissue (voxel density between -190 and -30) superficial to the abdominal skeletal muscles; VAT was defined as adipose tissue deep to the abdominal skeletal muscles; and TAT was defined as all cross-sectional adipose. Representative crosss-sectional images from this analytic approach are displayed in Figure 1. The ratio of VAT/SAT was calculated to approximate adipose distribution at the L4 level. Two-dimensional, cross-sectional skeletal muscle area and mean density were also measured at the mid-level of the L4 vertebra. Separate measurements were taken to assess the left psoas major muscle, right psoas major muscle, and total skeletal muscle. Skeletal muscle was identified by using a voxel density threshold of -29 HU to 150 HU and then applying a user-drawn ROI around the corresponding anatomical features. Total psoas area index (cm^2^/m^2^) was calculated by dividing total cross-sectional psoas muscle area at the L4 level (cm^2^) by the patient’s height squared (m^2^). Skeletal Muscle Index (SMI; cm^2^/m^2^) was calculated by dividing total cross-sectional skeletal muscle area at the L4 level (cm^2^) by the patient’s height at the time of surgery squared (m^2^).

The abdominal aorta-to-skin distance was measured between the center of the abdominal aorta and the anterior-most aspect of the superficial skin at the mid-level of the L3 vertebra as an approximation of vascular anastomotic distance from the incision. Mean Vertebral Body Density (HU) was measured at the transverse L1 vertebra level using a user-drawn, circular ROI, centered around the anterior third of the vertebral body.

The primary exposures were sarcopenia and visceral adiposity. Visceral adiposity was defined separately in men and women as the quartile of patients with the highest VAT/SAT ratio (≥0.84 in men and ≥0.51 in women). Sarcopenia was defined separately in men and women as the quartile of patients with the lowest SMI (<51.2 cm^2^/m^2^ in men and <43.1 cm^2^/m^2^ in women). Sensitivity analyses were conducted using alternative definitions of visceral adiposity and sarcopenia, including defining the criteria by median values in each sex (VAT/SAT ≥0.59 in men and ≥0.35 in women; SMI <57.8 cm^2^/m^2^ in men and <46.7 cm^2^/m^2^ in women) and by published thresholds from other studies (VAT/SAT of ≥1.325 in men and ≥0.710 in women; SMI <52.4 cm^2^/m^2^ in men and <38.5 cm^2^/m^2^ in women).

The primary outcomes were post-transplant patient and pancreatic graft survival rates. Pancreatic graft failure was defined as the occurrence of any of the following: death with a functioning graft, transplant pancreatectomy, relisting, or return to insulin use of >0.5 U/kg/day for at least three months duration. Secondary outcomes were kidney graft failure and PTDM. PTDM was defined as the resumption of oral or injectable hypoglycemia agents or insulin of any amount for at least three months duration. Subgroup analyses were separately performed on patients that received SPK and patients that received pancreas transplant alone (PTA), and on patients with T1DM and those with T2DM.

*4. Statistics*

Statistical analysis was performed with SAS software (Version 9.4, SAS Institute Inc., Cary, NC). Continuous variables were compared between groups using the Student’s t-test. Categorical variables were compared using χ2 or Fisher’s exact test, as appropriate. Post-transplant outcomes were examined using Kaplan-Meier curves and compared using a log-rank test for equality of survivor functions. Hazard ratios (HR) were calculated using Cox proportional hazards models.

| **Supplemental Table 1**. Demographics of pancreas transplant donors and recipients | | | |  | |  |
| --- | --- | --- | --- | --- | --- | --- |
| **Characteristic** | **Overall** | **Visceral Adiposity** | **No Visceral Adiposity** | | **p-value** | |
|  | n=204 | n=52 | n=152 | |  | |
| **Recipient Characteristics** | | | | | |  |
| Recipient age | 47.6 ± 10.1 | **50.7 ± 10.0** | **46.6 ± 10.0** | | **0.01** | |
| Recipient female | 36% (73) | 37% (19) | 76% (55) | | NA | |
| Recipient BMI | 26.9 ± 3.8 | 27.2 ± 4.3 | 26.7 ± 3.6 | | 0.44 | |
| Recipient race |  |  |  | | 0.07 | |
| Asian | 4% (9) | 8% (4) | 3% (5) | |  | |
| Black | 12% (24) | 8% (4) | 13% (20) | |  | |
| White | 82% (167) | 79% (41) | 83% (126) | |  | |
| Other | 2% (4) | 6% (3) | 1% (1) | |  | |
| Recipient T1DM | 71% (146) | **62% (32)** | **75% (114)** | | **0.046** | |
| Transplant type |  |  |  | | 0.43 | |
| SPK transplant | 80% (163) | 85% (44) | 78% (119) | |  | |
| Pancreas transplant alone | 20 % (41) | 15% (8) | 22% (33) | |  | |
| Total adipose tissue, cm^2^ | 372 ± 185 | **420 ± 198** | **356 ± 176** | | **0.03** | |
| Skeletal muscle, cm^2^ | 163 ±58 | 155 ± 43 | 165 ± 62 | | 0.21 | |
| SMI, cm^2^/m^2^ | 55 ± 19 | 53 ± 11 | 56 ± 21 | | 0.22 | |
| Skin-to-aorta distance, mm | 117 ± 28 | **132 ± 32** | **112 ± 25** | | **<0.01** | |
| L1 vertebral body density | 173 ± 54 | 165 ± 47 | 177 ± 57 | | **0.18** | |
| **Donor Characteristics** | | | | | |  |
| Donor age | 26.5 ± 12.1 | **23.3 ± 11.9** | **27.6 ± 25.6** | | **0.02** | |
| Donor female | 38% (78) | 35% (18) | 29% (60) | | 0.62 | |
| Donor BMI | 23.0 ± 3.9 | 23.1 ± 3.7 | 23.0 ± 3.7 | | 0.94 | |
| Donor race |  |  |  | | 0.45 | |
| Asian | 3% (7) | 6% (3) | 3% (4) | |  | |
| Black | 10% (21) | 6% (3) | 12% (18) | |  | |
| White | 79% (162) | 81% (42) | 79% (120) | |  | |
| Other | 8% (14) | 8% (4) | 7% (10) | |  | |
| Donation after circulatory death | 19% (39) | 13% (7) | 21% (32) | | 0.31 | |
| Pancreas donor risk index | 1.25 ± 0.5 | 1.22 ± 9.5 | 1.27 ± 0.5 | | 0.53 | |
| Pancreas cold ischemic time, hr | 12.8 ± 4.1 | 12.7 ± 3.6 | 12.9 ± 4.3 | | 0.72 | |
| **Short Term Outcomes** | | | | | |  |
| Hospital length of stay, days | 8.7 ± 4.4 | 9.1 ± 5.1 | 8.5 ± 4.2 | | 0.41 | |
| Readmission within 30 days | 56% (115) | 56% (29) | 57% (86) | | 1 | |
| Kidney delayed graft function in SPK patients | 11% (18) | 7% (3) | 13% (15) | | 0.4 | |
| BMI: body mass index; SMI: skeletal muscle index; SPK: simultaneous pancreas and kidney transplant; T1DM: type 1 diabetes mellitus; T2DM: type 2 diabetes mellitus | | | | | | |
